# Metagenomic Analysis of the Diversity of DNA Viruses in the Surface and Deep Sea of the South China Sea

**DOI:** 10.3389/fmicb.2019.01951

**Published:** 2019-08-23

**Authors:** Yantao Liang, Long Wang, Zengmeng Wang, Jiulong Zhao, Qingwei Yang, Min Wang, Kaiguang Yang, Lihua Zhang, Nianzhi Jiao, Yongyu Zhang

**Affiliations:** ^1^Key Laboratory of Biofuels, Shandong Provincial Key Laboratory of Energy Genetics, Qingdao Institute of Bioenergy and Bioprocess Technology, Chinese Academy of Sciences, Qingdao, China; ^2^College of Marine Life Sciences, Institute of Evolution and Marine Biodiversity, Ocean University of China, Qingdao, China; ^3^State Key Laboratory of Marine Environmental Science, Institute of Marine Microbes and Ecospheres, Xiamen University, Xiamen, China; ^4^Dalian Institute of Chemical Physics, Chinese Academy of Sciences, Dalian, China

**Keywords:** marine viruses, South China Sea, metagenomics, community structure, deep sea

## Abstract

A metagenomic analysis of the viral community from five surface and five deep sea water (>2000 m below the surface, mbs) samples collected from the central basin of the South China Sea and adjacent Northwest Pacific Ocean during July–August 2017 was conducted herein. We builded up a South China Sea DNA virome (SCSV) dataset of 29,967 viral Operational Taxonomic Units (vOTUs), which is comparable to the viral populations from the original Tara Ocean and Malaspina expeditions. The most abundant and widespread viral populations were from the uncultivated viruses annotated from the viral metagenomics. Only 74 and 37 vOTUs have similarity with the reported genomes from the cultivated viruses and the single-virus genomics, respectively. The community structures of deep sea viromes in the SCSV were generally different from the surface viromes. The carbon flux and nutrients (PO_4_ and NOx) were related to the surface and deep sea viromes in the SCSV, respectively. In the SCSV, the annotated vOTUs could be affiliated to the cultivated viruses mainly including *Pelagibacter* (SAR11) phage HTVC010P, *Prochlorococcus* phages (P-GSP1, P-SSM4, and P-TIM68), Cyanophages (MED4-184 and MED4-117) and *Mycobacterium* phages (Sparky and Squirty). It indicated that phage infection to the SAR11 cluster may occur ubiquitously and has significant impacts on bathypelagic SAR11 communities in the deep sea. Meanwhile, as *Prochlorococcus* is prominently distributed in the euphotic ocean, the existence of their potential phages in the deep sea suggested the sedimentation mechanism might contribute to the formation of the deep sea viromes. Intriguingly, the presence of *Mycobacterium* phages only in the deep sea viromes, suggests inhabitance of endemic viral populations in the deep sea viromes in the SCSV. This study provided an insight of the viral community in the South China Sea and for the first time uncovered the deep sea viral diversity in the central basin of the South China Sea.

## Introduction

Viruses are the most numerous, ubiquitous and diverse organisms in the aquatic environment. They play an essential role in regulating microbial fatality, community, and functions, and impact the microbial genetic diversity through the horizontal gene transfer (Wommack and Colwell, 2000; Weinbauer, 2004; Suttle, 2005, 2007). By controlling microbial community and inducing the release of organic matters from host cells, viruses significantly impact the marine biogeochemical cycles (Suttle, 2005) and contribute to the sequestration of CO_2_ through “microbial carbon pump” and “biological pump” (Jiao et al., 2010; Guidi et al., 2016).

Though viruses are of great significance in the marine ecosystems, their diversity and variability are still not clearly understood as compared to that of the bacterial communities. This is mainly because of lacking universal gene markers for investigating viral communities and the relatively recent development and application of culture-independent high-throughput sequencing methods (Breitbart et al., 2002; Brum et al., 2015; Aylward et al., 2017). With the rapid progress of the high-throughput sequencing technology and bioinformatic analysis, metagenomics has become an essential and powerful method to better understand viral community structure, diversity, and variability than the original culture-dependent and gene markers dependent methods. Mainly it can be easily used to compile and annotate the complex, pristine ecological viral genomes (Angly et al., 2006; Culley et al., 2006; Hurwitz and Sullivan, 2013; Paez-Espino et al., 2018; Roux et al., 2018). During the last two decades, the viral communities have been resolved from more and more different marine environments through the metagenomics, particularly through several great expedition and datasets like the Global Ocean Sampling Expedition, Pacific Ocean Virome, Tara Ocean Expedition, Malaspina expedition, Tara Oceans Polar Circle (TOPC) expedition and so on (Angly et al., 2006; Steward and Preston, 2011; Williamson et al., 2012; Hurwitz and Sullivan, 2013; Brum et al., 2015; Roux et al., 2016; Gregory et al., 2019). Recently, the Global Ocean Viromes 2.0 (GOV 2.0) dataset from 145 marine viral metagenomic samples identified a total of 195,728 viral populations, which is about 12-fold of viral populations from the original Tara Ocean and Malaspina expeditions (Brum et al., 2015; Roux et al., 2016; Gregory et al., 2019). However, comparing with the virome information in the euphotic zone, our knowledge about the deep seas is still limited, especially in the Northwest Pacific Ocean (Winter et al., 2014; Mizuno et al., 2016; Gregory et al., 2019).

The South China Sea (SCS) is an immense marginal sea in the Northwest Pacific Ocean and connects the Pacific Ocean and the Indian Ocean through the complex western boundary currents systems (Hu et al., 2015). The SCS located in the Western Pacific Warm Pool that potentially influence global marine ecosystems and climate change. Nonetheless, there are still few reports on viral diversity in the South China Sea. Till date, there have only been two studies about the viral diversity using the gene markers (DNA polymerase gene and g23 gene, respectively) in the SCS (Huang et al., 2010; He et al., 2017). The viral community structure of cyanophage in one surface sample of the central SCS was distantly discriminated from the samples from other oceans (Huang et al., 2010), suggested that the viral community structure and diversity of SCS might contain unique features. Currently, there is still not metagenomic report of the viral community structure and diversity in the South China Sea, especially in the deep sea (>1,000 m below the surface, mbs), which is awaiting in-depth investigation to unveil their unknown ecological characteristics.

To illustrate the viral community and diversity in the deep sea of South China Sea and to compare the differences of virome information between surface and deep sea, we present a DNA viral metagenomic dataset, including 10 seawater samples from the surface and deep sea layers (from about 2,000 to 3,500 mbs) of five stations in the central South China Sea and adjacent Northwest Pacific Ocean. This study will present the first metagenomic insight on the viral diversity, community structure and the differences among surface and deep sea seawater samples in the central South China Sea.

## Materials and Methods

### Sampling and Analysis of Environmental Factors

**FIGURE 1:**
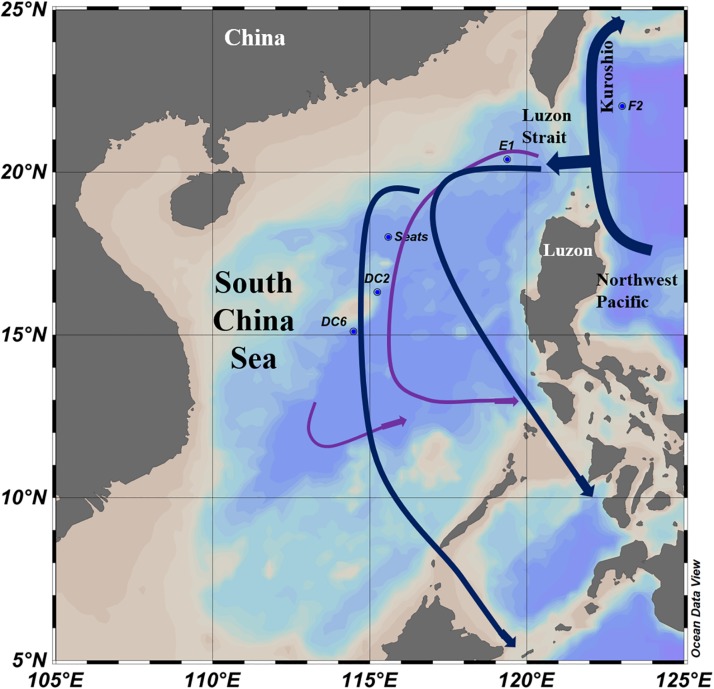
Sampling stations and water currents (modified from Wang et al., 2011) in the South China Sea. The surface water currents (Blue lines) were revised from Hu et al. (2015). The deep sea water currents (Purple lines) in the central basin were revised from Wang et al. (2011).

Ten viral metagenomic seawater samples (five surface and five deep sea deeper than 2,000 m) were collected from four stations (E1, SEATS, DC2, DC6) in the central South China Sea and one station (F2) in the Northwest Pacific Ocean adjacent to the South China Sea during July–August 2017 (Figure 1). Seawater samples (240 L) were collected using Niskin bottles fitted on a rosette frame which also equipped the SBE-9 plus CTD sensors (SBE 911; Sea-Bird Electronics) for temperature, salinity, and depth. Hundred milliliter sub-samples were collected from each site to determine concentrations of nutrient and dissolved organic carbon (DOC). Nutrient concentrations PO_4_, NO_2__,_ and NOx (NO_2_ + NO_3_) were investigated using an onboard nutrient auto-analyzer (SKALAR SAN plus, Netherlands). The detection limits for PO_4_, NO_2__,_ and NOx were 0.03, 0.01, and 0.03 μM, respectively (Han et al., 2012). Unfiltered DOC samples were stored in precombusted EPA vials at −20°C until further analysis. The TOC concentration was measured using a Shimadzu TOC-V analyzer according to Wu et al. (2015).

The carbon fluxes of each sample were collected from the primary production for the surface samples and the vertical flux (*F*_z_; g C m^–2^ yr^–1^) of particulate organic carbon (POC) for the deep sea samples. The *F*_z_ was estimated according to Antia et al. (2001) and Yokokawa et al. (2013):

(1) F=z0.1×(PP)×1.77(Z)-0.68

*F*_z_ related to primary production (PP; g C m^–2^ yr^–1^) for depth (Z; m) greater than 100 m. For each station, the PP during July 2017 was acquired from the custom products of the Ocean Productivity website^1^ based on the Epply Vertically Generalized Production Model from MODIS/Aquar data (Behrenfeld and Falkowski, 1997).

### Preparation of Viral Concentrates, DNA Extraction, and Sequencing

The seawater samples (240 L) were immediately filtered through a 300 mm diameter cellulose membrane with a 3 μm pore size, and then re-filtered the filtrate through a 0.22 μm membrane, to remove the bigger organisms, such as zooplankton, phytoplankton, and bacteria. Free viruses in the final filtrate were concentrated to a volume of 500 mL by the large-scale Tangential Flow Filtration (membrane package with a total surface area of 0.5 m^2^: Pellicon^®^ 2 Cassette, Biomax^®^ 50 kDa; polyethersulfone) (Sun et al., 2014). After concentrating sample each time, the filtration membrane cassettes were cleaned by rinsing with ample virus-free deionized water, followed by cleaning with 0.1 N NaOH for at least 30 min. The concentrated viral samples were stored in the black polycarbonate bottle and then stored at 4°C until being further processed (Angly et al., 2006; Thurber et al., 2009; Cai et al., 2016).

Just before the analysis, the viral concentrates were re-filtrated through a 0.22 μm filter to take away any remaining cellular microorganisms or aggregation. The filtrates were treated with 2 ng ⋅ L^–1^ DNase I at room temperature for 1 h to remove the free DNA. After digestion, NaCl (final concentration: 1 M) was added and incubated at 4°C for 1 h. The filtrates were concentrated using polyethylene glycol (PEG-8000) precipitation (10% w/v) and incubated at 4°C in the dark for 24 h. The mixed samples were centrifuged at 10,000 × *g* for 60 min at 4°C, and the pellets were resuspended in SM buffer. Afterward, the virome samples were purified by CsCl gradient ultra-centrifugation (gradient-density: 1.5 g ⋅ mL^–1^, 200,000 × *g*, 8 h, 4°C; CP-100WX). The purified viral particles were collected and dialyzed three times in SM buffer using 30 kDa super-filters (UFC5030) for DNA extraction. The viral DNA was obtained using the phenol/chloroform/isoamylol method and stored at -80°C until sequencing (Thurber et al., 2009). High-throughput sequencing of the original viral DNA was carried out by Novogene (Beijing, China) using Illumina NovaSeq 6000 (pair-end sequencing, 2 × 150 bp).

### Metagenomic, Genomic, and Function Analyses

Only the high-quality reads were picked from the raw reads giving 30–51 million (clean data rate >0.90) 150 bp paired-end reads. The paired-end reads were then segregated by implying the following conditions: (1) having more than 10% N; (2) were of low quality (40% reads length, Q ≤ 5); (3) with the adapter. Quality-filtered reads were assembled using metaSPAdes (version 3.12.0) (Nurk et al., 2017). The contigs with lengths of less than 300 bp were filtered out. The assembled contig data were analyzed using VirSorter V2 for the viral predictions (Roux et al., 2015). Only the sure (category 1) and somewhat sure (category 2) phage contigs and prophages (category 4 and category 5) were considered as viral contigs and used for further analysis. A short sketch of the 10 viromes is shown in Table 1. The average abundance was calculated as the transcripts (gene) per million reads mapped (TPM) using Salmon (Patro et al., 2017).

**TABLE 1:** Data summary of 10 viral metagenomic samples in the South China Sea and adjacent Northwest Pacific Ocean.

**Sample name**	**Raw reads**	**Clean bases (G)**	**Q20 (%)**	**Q30 (%)**	**GC content (%)**	**Viral contigs (>300 bp)**	**vOTUs**
F2_5	31,114,321	9.32	98.15	94.17	42.95	10, 592	5, 987
F2_3500	46,752,263	14.02	96.80	91.35	54.08	5, 803	3, 051
E1_5	30,307,542	9.05	96.87	91.05	39.35	18, 395	10, 407
E1_3000	39,497,164	11.83	96.75	91.05	51.86	6, 997	3, 988
SEATS_5	36,978,668	11.08	97.61	92.71	37.37	23, 257	13, 001
SEATS_3000	51,175,713	15.34	97.49	92.84	48.92	11, 150	6, 732
DC2_5	37,768,566	11.31	96.66	90.62	37.25	20, 810	11, 677
DC2_2000	47,358,488	14.20	97.66	93.27	50.79	8, 333	4, 994
DC6_5	33,065,273	9.88	97.07	92.15	39.36	10, 989	10, 124
DC6_2000	54,758,166	16.41	98.19	94.36	52.28	5, 626	2, 907

The taxonomic annotation and potential hosts of the viral contigs from the VirSorter 2.0 were blasted to the Integrated Microbial Genome/Virus (IMG/VR) system v.2.0^2^ dataset using the routine parameters (Paez-Espino et al., 2018). The open reading frames (ORFs) were predicted for each viral contig through Prodigal (Noguchi et al., 2008).

The functional contents of the 10 SCSV samples and the deep sea unique viral contigs were further characterized using Meta Genome Rapid Annotation using Subsystem Technology (MG-RAST) (Meyer et al., 2008) (with MG-RAST accession number 4839906.3, 4839904.3, 4839922.3, 4839908.3, 4839912.3, 4839916.3, 4839914.3, 4839910.3, 4839920.3, 4839918.3, and 4840005.3, respectively), an online metagenome annotation service^3^. The viral contigs processed by MG-RAST were compared to the SEED Subsystems database using a maximum *E*-value of 10^–5^, a minimum alignment length of 15, and a minimum identity of 60%.

### Phylogenetic Analysis

The phage terminase large-subunit domain (*Ter*L) and family B DNA polymerase (DNA polB), which were present in phages of the order Caudovirales (Terminase_6, PF03237) and eukaryotic and cyanobacteria viruses respectively (Chen and Suttle, 1995; Koonin et al., 2015; Roux et al., 2017), were utilized to establish the phylogenetic tree. The TerL and DNA polB sequences were dereplicated at the 97% nucleotide level using cd-hit (Li and Godzik, 2006). The TerL and DNA polB sequences from the SCSV virome genes were screened by the DOE-JGI Metagenome Annotation Pipeline and compared to the viral RefSeq database using BLASTP (*E*-value < 10^–5^) to recruit relevant reference sequences. All sequences were aligned at the amino acid level using MUSCLE (Edgar, 2004) (using default parameters), manually inspected and trimmed as necessary. The maximum likelihood (ML) tree with 1000 bootstraps was constructed using the program FastTree (v2.1.10) (Price et al., 2010) using a JTT + CAT model and an estimation of the gamma parameter. The phylogenetic tree was visualized and displayed using iTOL (Interactive Tree of Life) (Letunic and Bork, 2016).

### Statistical Analyses

The cluster analysis and non-metric multidimensional scaling (NMDS) analysis of the DNA viral communities were performed using PRIMER v7 (PRIMER E, Ltd., United Kingdom) (Relative abundance of viruses in each sample represent the biont number). The difference analysis, venn diagram and heatmap of the viral clusters between the surface and deep sea were created using the free online platform of Majorbio I-Sanger Cloud Platform^4^. Canonical correspondence analysis (CCA) was performed in R v. 3.5.1 (R Development Core Team) using CCA and RDA functions from the “vegan” package v2.5-2 (Oksanen et al., 2018) to interrogate the relationships between viral clusters and environmental variables. A matrix of the total viral Operational Taxonomic Units (29,967 vOTUs) was processed for factor analysis. A total of nine environmental variables were used to assess the variation of viral species, including longitude, latitude, depth, temperature, salinity, NO_x_, PO_4_-P, total organic carbon (TOC) and carbon flux. All variables were logarithmically (base 10) converted before CCA to reduce the influence of extreme values on ordination scores and to normalize data distribution.

### Accession Number

All the viral reads data in this study were submitted to the NCBI Sequence Read Achieve (SRA). The SRA accession number: PRJNA535364.

## Results and Discussion

### The South China Sea Viromes (SCSV) Dataset and Contig Assembly

The 10 South China Sea Viromes (SCSV) data set contains 122 Gb of sequences from five surface and five deep sea (>2000 m) samples in the central basin of the South China Sea and the adjacent Northwest Pacific Ocean (Figure 1 and Table 1). The SCSV data set offers the first glimpse of surface and deep sea viral communities of the South China Sea, which is the largest marginal sea located at the Northwest Pacific Ocean. The SCSV samples were filtered through 0.22 μm pore–size filters, and then the viruses were concentrated using the TFF method. These steps are one of the conventional techniques to collect the virome samples, which would have removed most of the prokaryotic and eukaryotic cells, and would also have excluded most of the viruses larger than 0.22 μm (Brum et al., 2015; Gong et al., 2018). The SCSV viral concentrates were purified using DNase digestion and CsCl density gradients to reduce contamination by non-viral DNA (Hurwitz and Sullivan, 2013). The extracted viral DNA was sequenced directly without the amplification which excludes the bias against single-stranded DNA (ssDNA) viruses caused by the amplification treatment (Duhaime and Sullivan, 2012; Marine et al., 2014).

In the SCSV data set, the assembled contig data was analyzed for the viral predictions using VirSorter V2. A total of 121,952 viral contigs longer than 300 bp (of which 99,791 viral contigs were ≥1,500 bp) were predicted and ranged from 5626 at DC6_2000 to 23,257 at SEATS_5. The viral contigs were blasted against the IMG/VR 2.0 dataset to classify the viral contigs as the vOTUs and then classify vOTUs that corresponding to the viral populations in the GOV 2.0 data set (Gregory et al., 2019). A total of 29,967 vOTUs were predicted (of which 27,249 vOTUs were ≥1,500 bp, and 5,857 vOTUs were ≥10 kb) and ranged from 2,907 vOTUs at DC6_2000m to 13,001 vOTUs at SEATS_5m, which is comparable to the viral populations from the original Tara Ocean and Malaspina expeditions (15,280 viral populations which are defined as viral contigs have ≥95% ANI across its members) (Roux et al., 2016). The taxonomic analysis of the viral populations based on virome data remains arduous (Brum and Sullivan, 2015; Brum et al., 2015). Only recently, the establishment and combination usages of the VirSorter 2.0 and IMG/VR 2.0 data sets have greatly improved the assembly of large contigs (up to 100 kb) and aided the taxonomic analysis of viral populations at the genome-level and viral-cluster-level (Roux et al., 2015; Paez-Espino et al., 2018). Most of the viral contigs of SCSV (117,161 viral contigs, 90.9% of total viral contigs) predicted by the VirSorter 2.0 could be taxonomically classified as vOTUs using the IMG/VR 2.0 data set, indicating the feasibility and high-efficiency of the taxonomic classification using the IMG/VR 2.0 data set.

### The Genetic Diversity of vOTUs in the SCSV

**FIGURE 2:**
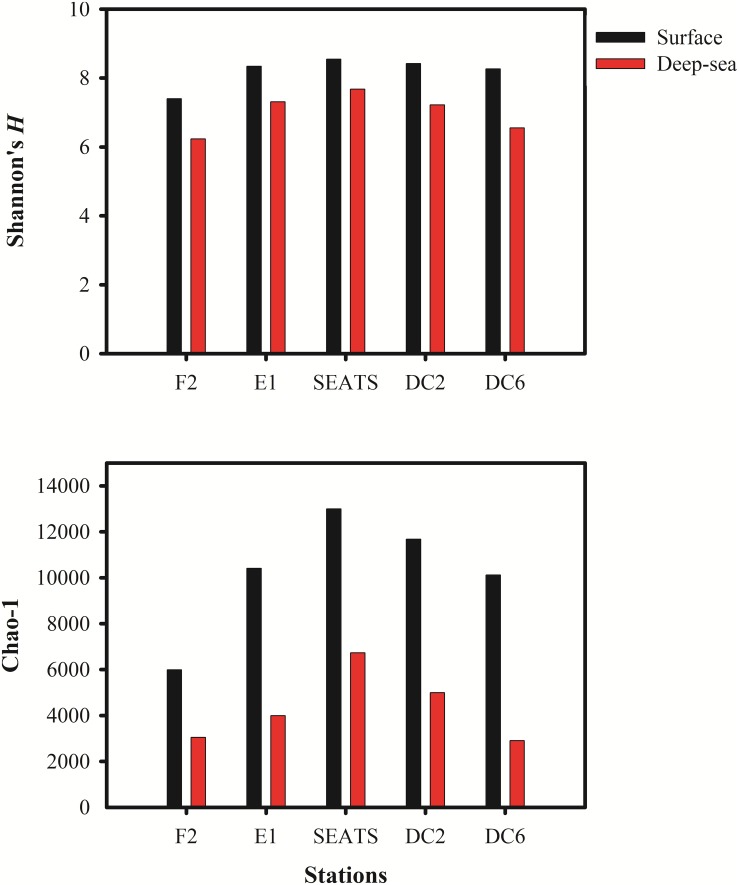
The Alpha diversity indexes in South China Sea viromes (SCSV) data set. **(A)** Shannon’s diversity *H*’ calculated from the relative abundance of viral Operational Taxonomic Units (vOTUs) for each sample. **(B)** Chao 1 diversity calculated from relative abundances of vOTUs for each sample. The relative abundance is computed for each sample as the number of the transcripts per million reads mapped (TPM).

The Shannon’s *H’* and Chao I diversity indexes of the vOTUs in each sample were shown in Figure 2. The deep sea viromes were less diverse (the Shannon’s *H’* ranged from 6.23 at F2_3500 to 7.68 at SEATS_3000, and Chao I ranged from 2907 at DC6_2000 to 6732 at SEATS_3000) than the surface viromes (the Shannon’s *H’* ranged from 7.40 at F2_5 to 8.54 SEATS_5 and Chao I ranged from 5987 at F2_5 to 13,000 at SEATS_5, respectively). The diversity of vOTUs in the F2 station located at the adjacent Northwestern Pacific Ocean was lower than that in the stations within the South China Sea (Figure 2). The high primary production (PP) at the SEATS station (473.4 mg C m^–2^ day^–1^) and the low PP at the F2 station (387.6 mg C m^–2^ day^–1^) might be the reason of the diversity patterns of vOTUs in the SCSV data set.

### Taxonomic Composition and Potential Hosts of Viral Communities in the SCSV

**FIGURE 3:**
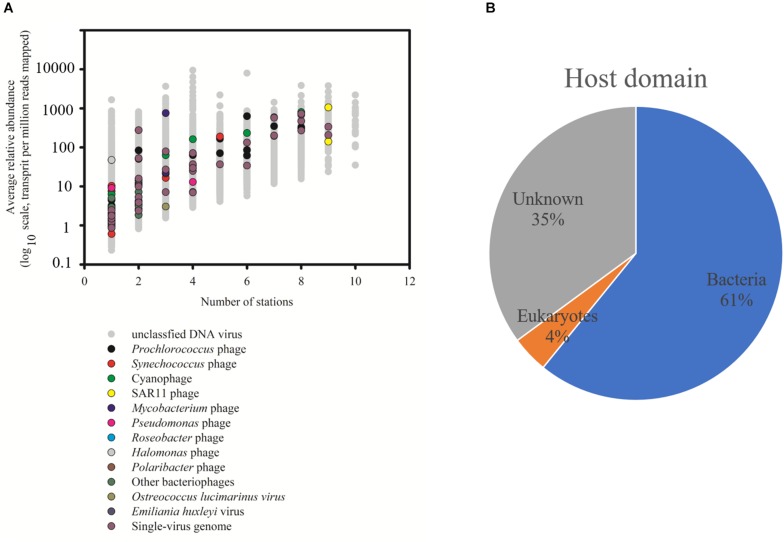
Taxonomic affiliation of viral Operational Taxonomic Units (vOTUs) of South China Sea viromes (SCSV) sorted by distribution and average abundance **(A)**. The potential host domains of viral contigs of SCSV **(B)**. The reference genome of vOTU from the cultivated viruses and single-virus genomics were shown. As in Figure 2, the relative abundance (y axis) is computed for each sample as the number of the transcripts per million reads mapped (TPM). Here, the relative abundance of a vOTU is defined as the average abundance of this vOTU across all samples.

The relative abundance of 29,967 vOTUs in the SCSV was estimated using the IMG/VR 2.0. Only 20 vOTUs were observed across all the 10 samples, and 3,893 vOTUs (13.0%) were commonly observed across > five samples. 13,001 vOTUs (43.4%) were endemic to one sample, which is different from the results of Tara Ocean expedition (15% of viral populations were observed at only one station) (Brum et al., 2015). The high percent of the endemic vOTUs in the SCSV might reflect the high diversity of viral communities in the South China Sea. Only 74 of 29,967 vOTUs (0.25%) from IMG/VR 2.0 could be affiliated to cultivated reference viruses, which reflects the shortage of reference viral genomes in the databases (Brum et al., 2015; Roux et al., 2016). These cultivated viruses include the viruses infecting the dominant and widespread hosts SAR11, *Prochlorococcus*, *Synechococcus*, *Roseobacter*, *Pseudomonas*, *Pseudoalteromonas*, *Mycobacterium*, *Halomonas*, *Polaribacter*, *Ostreococcus lucimarinus*, *Emiliania huxleyi*, etc. (Figure 3A; Kang et al., 2013; Zhao et al., 2013; Huang et al., 2015; Liang et al., 2016). Additionally, there are 37 vOTUs could be affiliated to the uncultured viral genomes from the single-virus (35 vOTUs) or single-cell (2 vOTUs) genomics (Figure 3A; Martinez-Hernandez et al., 2017; Berube et al., 2018). However, the most abundant and widespread vOTUs observed in the SCSV were from the uncultivated viruses annotated from the viral metagenomics (Figure 3A). This indicates that most of the marine viruses are yet to be characterized even though more than 8,000 cultivated viral and single-virus genomes had been reported and deposited in the GenBank and represented several groups of the dominant microbial hosts (Kang et al., 2013; Zhao et al., 2013; Huang et al., 2015; Liang et al., 2016; Martinez-Hernandez et al., 2017; Berube et al., 2018). As most of the vOTUS affiliated to cultivated reference viruses are phages (Figure 3A) and the prominent predicted host domains using the IMG/VR 2.0 datasets are bacteria (62%, Figure 3B), the uncultured phages infecting prokaryotes might dominate the viromes in the SCSV.

### Comparison Between the Surface and Deep Sea Viromes in the SCSV

**FIGURE 4:**
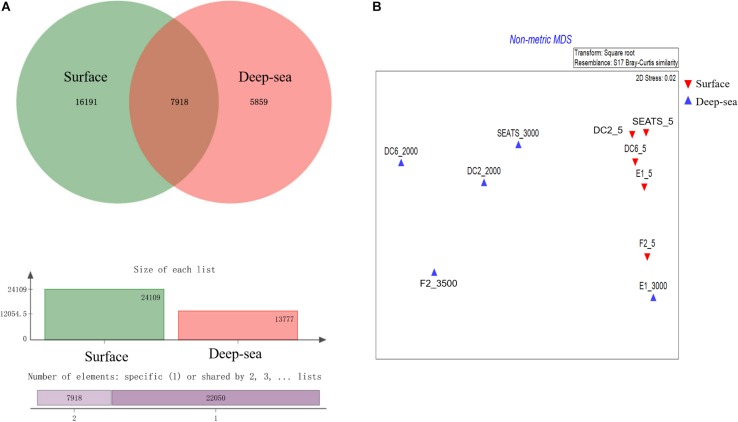
Comparisons of the surface and deep sea viromes in the South China Sea viromes (SCSV) data set. **(A)** The venn graph of the viral Operational Taxonomic Units (vOTUs) of the surface and deep sea viromes. **(B)** The Non-metric multidimensional scaling (NMDS) of SCSV based on the Bray-Curtis dissimilarity matrix calculated from the relative abundance of vOTUs from the SCSV data set.

**FIGURE 5:**
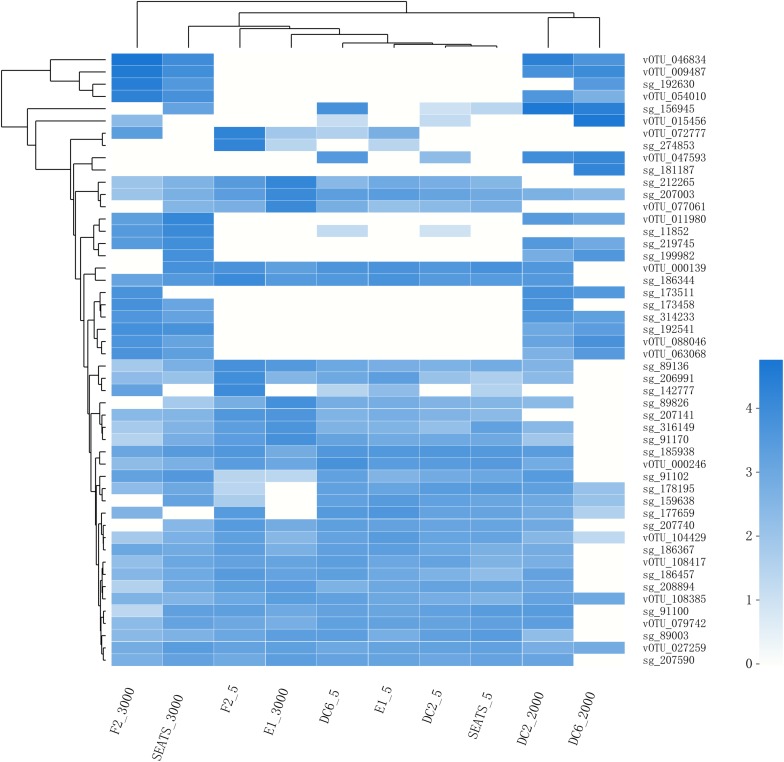
The heatmap of the relative abundance of the most abundant 50 vOTUs in the SCSV data set. As in Figure 2, the relative abundance is computed for each sample as the number of the transcripts per million reads mapped (TPM).

**FIGURE 6:**
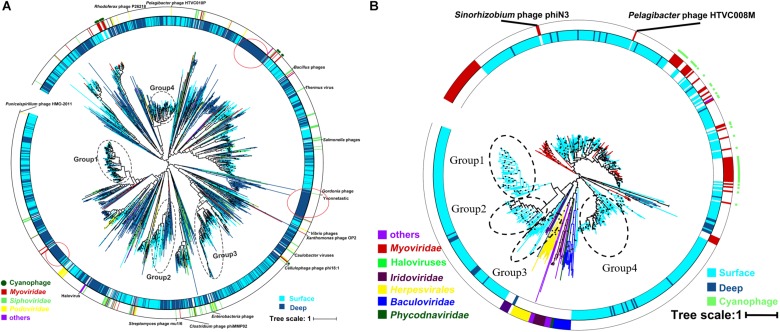
Phylogenetic tree of the viral contigs based on the terminase large-subunit domains **(A)** and family B DNA polymerase **(B)** in the South China Sea viromes (SCSV) data set. The maximum likelihood phylogenetic trees of the *Caudovirals* terminase large-subunit domains (PF03237, **A**) and family B DNA polymerase **(B)** are shown (1000 iterations, JTT + G model). Only bootstrap values >50% are indicated at the nodes of the tree, and bootstrap scores greater than 90% are indicated with a black dot. The original terminase and family B DNA polymerase sequences from the surface viromes in the SCSV are indicated by wathet blue, and the original sequences from the deep sea viromes in the SCSV are in dark blue. Reference sequences are marked (see color legend at the bottom).

The result of the venn analysis showed that 7918 vOTUs (26.4%) were observed at both the surface and deep sea viromes, while 16,191 (54.0%) and 5,859 (19.6%) vOTUs were endemic to the surface and deep sea viromes, respectively (Figure 4A). According to the results of NMDS assessed using Bray-Curtis dissimilarity distances, the surface and deep sea viromes (except the E1_3000m) could be generally divided into two groups, and the distances among the surface viromes were shorter than that of the deep sea viromes (Figure 4B). The heatmap of the relative abundance of most abundant 50 vOTUs showed that the distribution patterns of vOTUs in the deep sea viromes are different from that in the surface viromes (Figure 5). And the phylogenetic tree of the phage terminase large-subunit domains showed the presence of the several new viral groups in the SCSV (Groups 1–4) and the unique viral clusters in the deep sea viromes (Figure 6A). The result is comparable to the results from the GOV 2.0, which divided the surface and deep sea viromes in the tropical oceans into the bathypelagic (>2,000 m) and temperate and tropical epipelagic (0–150 m) ecological zones (Gregory et al., 2019). For the phylogenetic tree of the DNA polB sequences, several new viral groups in the SCSV were observed (Figure 6B), which is similar with the results from the TerL sequences (Figure 6A). However, the novel viral groups from the DNA polB in the deep-sea viromes were fewer than that from the TerL sequences (Figure 6), which might indicate the smaller proportion of eukaryotic and cyanobacterial viruses than bacteriophages in the deep-sea viromes of SCSV (Chen and Suttle, 1995; Koonin et al., 2015; Chénard et al., 2016; Roux et al., 2017). The results from the phylogenetic analysis using only one marker might not represent the acutal phylogenetic relationships among different viruses. In the future, the phylogenetic analysis using the whole viral genomes could provide more solid phylogenetic relationships among different viruses (Castelan-Sanchez et al., 2019).

Interestingly, the viral community in the deep sea virome of the E1 station (E1_3000m) was quite different from those of other deep sea viromes in the SCSV and closed to the surface water viromes (F2_5) in the NMDS biplot and heatmap (Fi Figures 4B, 5). The E1 station was at the west side of the Luzon Strait where the horizontal and vertical physical transportation is robust. The strong western boundary current (Kuroshio) transport seawater from the Northwest Pacific Ocean (around F2 station) into the northeast of the South China Sea (around E1 station) (Figure 1; Wang et al., 2011; Hu et al., 2015). The average velocity of flow across interfacial Ekman transport along isopycnal surfaces at their edges was frequently above 0.5 m s^–1^, which could transfer the surface water to the depth of ∼1,000 m within a few days (Yuan, 2002; Tian et al., 2009). Other vertical physical transportation like mesoscale eddies and solitons were active at the Luzon Strait (Yuan, 2002; Warnvamas et al., 2010). The combination of the vertical physical transportations could probably transport the *Prochlorococcus* from surface waters into the mesopelagic zones (800 m) of the Luzon Strait (Jiao et al., 2014). We assumed that the strong horizontal and vertical physical transportation might be responsible for the existence of surface viruses of the adjacent Northwest Pacific Ocean (around F2 station) in the deep sea viromes of Northeast South China Sea (around E1 station). This is supported by the transportation of the surface viromes by the surface water currents in the Tara Ocean expedition (Brum et al., 2015).

**FIGURE 7:**
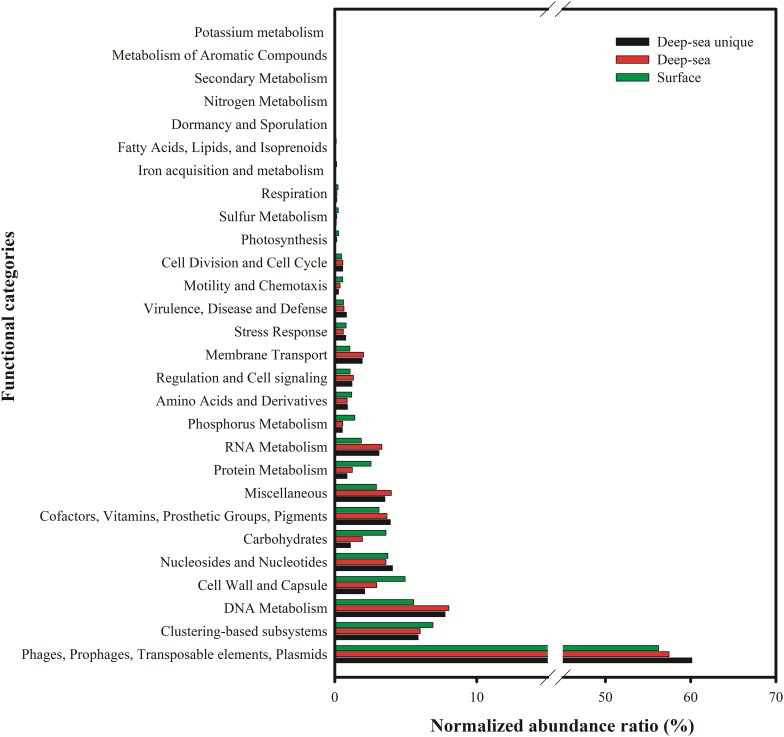
Functional analysis of viral contigs of surface, deep sea and deep sea unique contigs of the South China Sea viromes. The Coding Sequences (CDSs) were compared with the SEED database using subsystems in MG-RAST. The metabolic categorization is based on the sequences Best BLAST Hits in the SEED database curated subsystems (*E* < 10^–5^).

The putative functions of the viral contigs from the five surface viromes, five deep sea viromes and the deep sea unique viral contigs (5,859 contigs) were predicted using MG-RAST. Using the subsystems approach, nearly 58% (56.2–60.1%) of the annotated proteins were classified as “Phage, Prophage, Transposable elements, or Plasmids” (Figure 7). “DNA metabolism” (5.6–8.0%), “nucleosides/nucleotides” (3.6–4.1%), “Cofactors, Vitamins, Prosthetic groups, Pigment” (3.1–3.9%), “Miscellaneous” (2.9–4.0%), “Cell Wall and Capsule” (2.1–5.0%) were most commonly identified (>3%) and 5.9–6.9% of them were classified into “Clustering-based subsystems.” The deep sea unique viral contigs contain the highest proportion of functional categories of “Phage, Prophage, Transposable elements, or Plasmids” and the lowest proportions of “Cell Wall and Capsule,” “Carbohydrates,” “Protein metabolism,” and “Phosphorus metabolism” (Figure 7), which indicated the unique characteristics of deep sea viruses. It is likely that the deep sea viruses have less auxiliary metabolic activities compared to the viruses in the euphotic zone. As the functional analysis was limited to MGRAST, which is mainly limited to bacterial gene data rather than the viral and eukaryotic database, the functional analysis in the study might not represent the total functional properties of the viral communities in the SCSV. The usages of other platforms and strategies to analyze the virome (i.e., Virome, ViromeScan, Virus seeker, etc.) could improve the functional prediction of the SCSV in the future.

### Relationship Between Viral Community Structure and Environmental Factors

**FIGURE 8:**
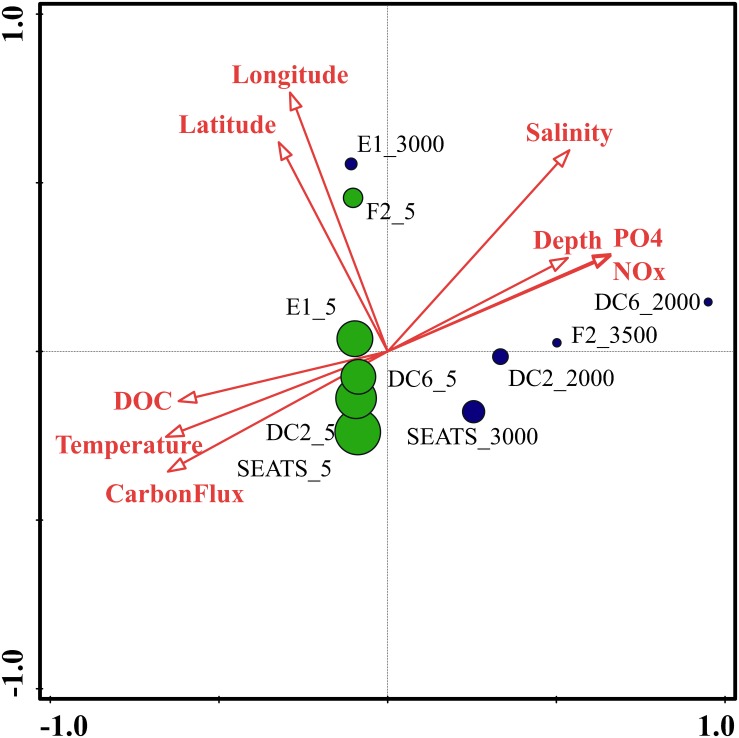
Canonical correspondence analysis of the relationship between the relative viral abundance of viral Operational Taxonomic Units (vOTUs) and environmental factors.

To identify the best predictor variables to explain the variation of the DNA viral community framework in the surface and deep sea of the South China Sea, multivariate regression analysis was used (Figure 8). The first CCA axis interpreted 31% of the total variability in the vOTUs, and the first two axes explained 48% of the total variability. The CCA demonstrated three clear groups of DNA viromes, including the surface viromes, the deep sea viromes and the Luzon physical transport viromes (F2_5 and E1_3000). Most of surface DNA viromes (E1_5, DC6_5, DC2_5, and SEATS_5) were firmly related to the carbon flux, temperature, and DOC. The deep sea DNA viromes (DC6_2000, F2_3500, DC2_2000, and SEATS_3000) were related to PO4, NOx, depth and salinity, while the Luzon vertical physical transport DNA viromes were most closely related to the longitude and latitude (Figure 8). The results suggest that the biogeography of surface and deep sea viral communities in the SCSV is structured by the environmental factors, which is similar with the results from the Tara Ocean, GOV 2.0 and Antarctic viromes (Brum et al., 2015; Roux et al., 2016; Gong et al., 2018; Gregory et al., 2019; Yang et al., 2019).

As the viruses require host cells to replicate, the biogeography trends of viruses usually follow the trends of the host community, which is mostly shaped by the environmental factors (Brum et al., 2015; Liang et al., 2017; Gregory et al., 2019). Hence, the relationship between viral communities and environmental factors in the SCSV might be the reflections of the relationships between host and viruses and between host communities and environmental factors. Interestingly, the close relationship between the surface virome of Northwest Pacific Ocean (F2_5) and deep sea virome of the Northeast South China Sea (E1_3000) might suggest the passive transport of viruses by not only the horizontal water currents which is testified by the Tara Ocean expedition (Brum et al., 2015), but also the vertical physical transportations (Figures 1, 4B, 5).

### The Possible Sources of the Deep Sea Viruses in the SCSV

**FIGURE 9:**
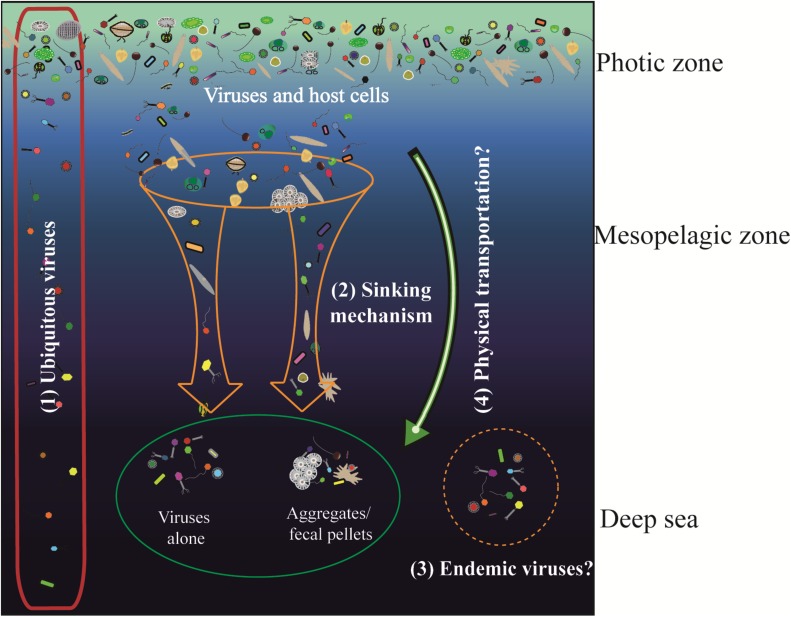
Schematic illustration of the mechanism of sources of the viruses in the deep sea (modified from Figure 7 of Guo et al., 2018). (1) Viruses were ubiquitous in the whole water column, e.g., SAR11 viruses; (2) viruses sank from euphotic zone into the deep sea; they could sink alone or within the aggregates/fecal pellets, e.g., *Prochlorococcus* phages and Cyanophages; (3) viruses might be endemic to the deep sea environments which infecting the deep sea unique host cells, e.g., *Mycobacterium* phages which were only observed in the deep sea viromes; and (4) the horizontal and vertical physical transportation processes might be involved in the fast transportation process to accelerate the sinking of viral and host cells.

According to the community structures and comparisons of the surface and deep sea viromes in the SCSV, we hypothesized several potential sources of the deep sea viromes (Figure 9). Firstly, some of the viruses are ubiquitous in the whole water column, which is coincident with their host organisms. For example, the SAR11 viruses (*Pelagibacter* phage HTVC010P) and SAR11 were abundant in both the surface and deep sea waters (Figures 3A, 5, 9; Zhao et al., 2013; Nunoura et al., 2015; Zhang et al., 2016). Secondly, the sedimentation mechanism might contribute to the formation of the deep sea viromes (Guidi et al., 2016). Several potential phages of autotrophic cyanobacteria were detected in the deep sea viromes, e.g., *Prochlorococcus* phages (P-GSP1, P-SSM4, and P-TIM68) and Cyanophages (MED4-184 and MED4-117) (Figures 3A, 5, 9). As *Prochlorococcus* are prominently distributed in the euphotic ocean (0–200 m), the existence of cyanophages in the deep sea viromes could be explained by the sedimentation mechanism, which is the most common and widely acknowledged theory accounting for the life of phytoplankton cells in the deep sea (Jiao et al., 2014; Agusti et al., 2015; Guo et al., 2018). The co-sinking of cyanophages with their host cells might also contribute to the existence of cyanophages in the viromes of the deep sea and sub-seafloor sediments (Guidi et al., 2016; Berube et al., 2018; Cai et al., 2019). Thirdly, a significant fraction of viruses is endemic to the deep sea viromes (Figures 4A, 5, 9), which might infect the deep sea dominant host organisms. For example, several vOTUs related to the *Mycobacterium* phages (Sparky and Squirty) were detected in the deep sea viromes and could probably infect the *Mycobacterium*, which was commonly detected in the deep sea waters and sediments (Lu et al., 2011). Fourthly, the similarity of viromes between E1_3000 and F2_5 might suggest that the passive transport of viruses by the horizontal and vertical physical transportations contributed to the formations of the deep sea viromes (Figures 1, 4B, 5, 9). In the future, intensive sampling and studies of viromes in the areas between Northwestern Pacific Ocean and northeast of South China Sea could give robust verification about the hypothesis that viral communities might be used to trace water sources.

## Conclusion

This is the first study to explore and reveal the main characteristics of both the surface and deep sea viromes in the South China Sea using the metagenomic analysis. The most dominant and widely spread viral populations in the SCSV were not similar with the reported genomes from the cultivated viruses and the single-virus genomics, which reflected the superiority of viral metagenomics in revealing the biogeography patterns of natural viromes and their relationship with environmental factors. The community structures of deep sea viromes were different from the surface viromes, though one deep sea virome (E1_3000) in the Northeast South China Sea is similar with one surface virome in the Northwest Pacific Ocean, which suggested the possible passive transport of marine viruses by the horizontal and vertical physical transportations. The presence of viruses in the deep sea waters may benefit from ubiquitous viruses, sinking mechanism, endemic living on the deep sea host communities and passive transport through physical transportation. In the future, it will be obligatory to evaluate the host community structure simultaneously, to verify the notionally theorized linkage among viruses, host communities and environmental conditions. The comparisons between SCSV and other studies, such as Tara Ocean, Malaspina and etc. could illustrate the unique feature of SCSV in the future.

## Data Availability

The datasets generated for this study can be found in NCBI, PRJNA535364.

## Author Contributions

YL, YZ, and NJ designed this study. YL, ZW, and JZ performed the experiments. Data were analyzed by YL, LW, and QY. YL and YZ wrote the manuscript. LW, MW, KY, LZ, and NJ contributed to writing by providing suggestions and helping with the revisions. All authors reviewed and approved the final version of the manuscript.

## Conflict of Interest Statement

The authors declare that the research was conducted in the absence of any commercial or financial relationships that could be construed as a potential conflict of interest.

## Footnotes

1 http://www.science.oregonstate.edu/ocean.productivity/

2 https://img.jgi.doe.gov/vr/

3 http://www.mg-rast.org/

4 www.i-sanger.com
